# Low Temperature Characteristics of Hydrogen Storage Alloy LaMm-Ni_4.1_Al_0.3_Mn_0.4_Co_0.45_ for Ni-MH Batteries

**DOI:** 10.3390/ma12244220

**Published:** 2019-12-16

**Authors:** Malgorzata Karwowska, Karol J. Fijalkowski, Andrzej A. Czerwiński

**Affiliations:** 1 Faculty of Chemistry, University of Warsaw, ul. Pasteura 1, 02-093 Warsaw, Poland; m.karwowska.chem@gmail.com (M.K.); aczerw@chem.uw.edu.pl (A.A.C.); 2 Centre of New Technologies, University of Warsaw, ul. Banacha 2c, 02-097 Warsaw, Poland

**Keywords:** Ni-MH batteries, AB_5_ alloy, hydrogen sorption, low temperature performance, Limited Volume Electrode (LVE)

## Abstract

Nickel hydride batteries (Ni-MH) are known of their good performance and high reliability at temperatures below 0 °C, which is significantly dependent on electrolyte composition. Here we present the low temperature characteristics of pristine AB5-type alloy, LaMm-Ni_4.1_Al_0.3_Mn_0.4_Co_0.45_, determined in various alkali metal hydroxide solutions. We found that the combination of KOH with NaOH showed a significant effect of enhancement of low temperature performance of the electrode material and diffusion of hydrogen in the alloy. This 6M binary mixed NaOH/KOH electrolyte, comprising 4M KOH component and 2M NaOH component, made it possible to maintain 81.7% and 61.0% of maximum capacity at −20 °C and −30 °C, respectively, enhancing the hydrogen storage properties of the alloy after reheating to room temperature.

## 1. Introduction

Nickel-metal hydride batteries (Ni-MH) are widely used electrochemical power sources. Although recently displaced by modern Li-ion batteries in diverse applications, Ni-MH batteries have proved their utility for powering various mobile and stationary devices, including Hybrid Electric Vehicles and large-scale emergency power systems [1,2,3,4,5]. Since the use of nickel-cadmium batteries is prohibited for standard applications [6], Ni-MH systems remain one of the most reliable power sources for working at low temperatures below 0 °C, where the electrochemical performance of all battery systems significantly decreases [7]. Commercially available Ni-MH batteries are designed to operate above −20 °C, because at that temperature they can already maintain less than 60% of their starting capacity according to producers’ manuals [8,9,10], although some tests have reported higher values [11]. For comparison, the capacity of nickel-cadmium batteries at −30 °C decreases to ca. 55%, while lead-acid batteries maintain only ca. 30% of their capacity at this temperature [12]. For many years, standard lithium-ion batteries have been able to retain only up to ca. 20% of their capacity at −30 °C, but recent optimization of the electrolyte composition enabled the enhancement of the low temperature performance of Li-ion batteries, which can retain up to 60% capacity at −30 °C, albeit with relatively low absolute capacities [13,14,15].

Electrochemical performance of Ni-MH batteries is determined by hydrogen diffusion rate from the bulk of the electrode to its surface [16], the kinetics of charge transfer reaction [16], and the mechanical stability of the negative electrode material [17,18,19] and its chemical corrosion [18,20,21,22]. However, at temperatures below −20 °C, additional factors should be taken into consideration when discussing the mechanism of diminishing performance of Ni-MH batteries, i.a., diminished conductivity and increased viscosity of the electrolyte, and emergence of local concentration gradients [11]. 

Various attempts to increase the performance of Ni-MH batteries at low temperatures have been tested and discussed, e.g., Ye and Zhang showed the positive impact of boron doping of the AB_5_ alloy on the kinetic characteristics of the electrode [23] which can significantly enhance its low temperature capacity even at −35 °C [7]. Also, modifications of the battery mantle and active heating of the battery pack have been proposed [11]. Unfortunately, most low temperature studies only consider 6M KOH as an optimum electrolyte and do not discuss possible modifications to its composition.

In this paper, we present a study on the impact of electrolyte composition on low temperature performance of AB_5_-type hydrogen storage alloy (LaMm-Ni_4.1_Al_0.3_Mn_0.4_Co_0.45_) used as a negative electrode in Ni-MH batteries. The main goals of this work were as follows: (i) systematic study of the capacity of the AB_5_-type alloy at low temperatures in various alkali metal hydroxide solutions; (ii) correlation of the capacity of the alloy observed at low temperatures with hydrogen diffusion coefficient of the alloy; and (iii) attempt to optimize the composition of the electrolyte among simple binary alkali metal hydroxide mixed solutions.

## 2. Materials and Methods

### 2.1. Working Electrode

LaMm-Ni_4.1_Al_0.3_Mn_0.4_Co_0.45_ hydrogen storage alloy (AB_5_-type) was used as a working electrode during the presented experiments. The alloy was prepared using lanthanum-rich mischmetal (LaMm) comprising 60% lanthanum, 26% cerium, 10% neodymium and 4% praseodymium, according to PXRD Rietveld refinement [24] (Figures S1–S5; Tables S1 and S2).

The general characteristics of the alloy under ambient conditions (0–30 °C) were reported in our previous reports [24,25,26,27]. The alloy crystallises in hexagonal P6/mmm Cu_5.4_Yb_0.8_ lattice [28], which is a modification of the CaCu_5_-type crystal structure adopted by parent LaNi_5_ [29], with unit cell: a = b = 5.0079(5) Å, c = 4.0521(4) Å, V = 88.007(16) Å^3^ (ICSD number 427242) [24] (Figure S2). The alloy powder consists of quasi spherical (Φ = 1.146) micrometre-scale grains (ca. 50 μm) with relatively large surface area (606 cm^2^/g, 1539 cm^2^/cm^3^) [24] (Figure S6, Table S3). The maximum theoretical hydrogen capacity of the alloy determined from PCT measurements is equal to 289 mAh/g, which corresponds to 4.7 at.H/f.u. [26], which is slightly lower than the maximum capacity of neat LaNi_5_ (5.5 at.H/f.u.) [17] (Figures S7 and S8). The equilibrium pressure of hydrogen sorption of the investigated alloy is relatively low at room temperature (0.7 atm) [26], which is significantly lower than the equilibrium pressure of neat LaNi_5_ (1.7 atm) [17]; thus, hydrogenation of the earlier is more favourable. The alloy undergoes corrosion processes (selective oxidation) in hydroxide solutions resulting in formation of characteristic needle-shaped Mm(OH)_3_ crystals and nickel metal aggregates [22,27].

The Limited Volume Electrode (LVE) method [30,31,32,33] was adapted to investigate electrochemical behaviour of neat LaMm-Ni_4.1_Al_0.3_Mn_0.4_Co_0.45_ alloy (Figure S1). Pellet electrodes were prepared by high pressure treatment (20 MPa) of the pristine alloy placed between two inert golden mesh current collectors (Good Fellow, 99.99% Au, 0.25 mm) in the same procedure, as described previously [24,25,26,27,34]. No additives or binding materials (e.g., metal powders, graphite, PTFE, PVA) were used, as they could affect the electrochemical characteristics of the investigated material [1,25,35,36,37,38]. Thus, the results presented in this study may differ from other reports, where the active material being investigated was not pristine.

**Figure 1:**
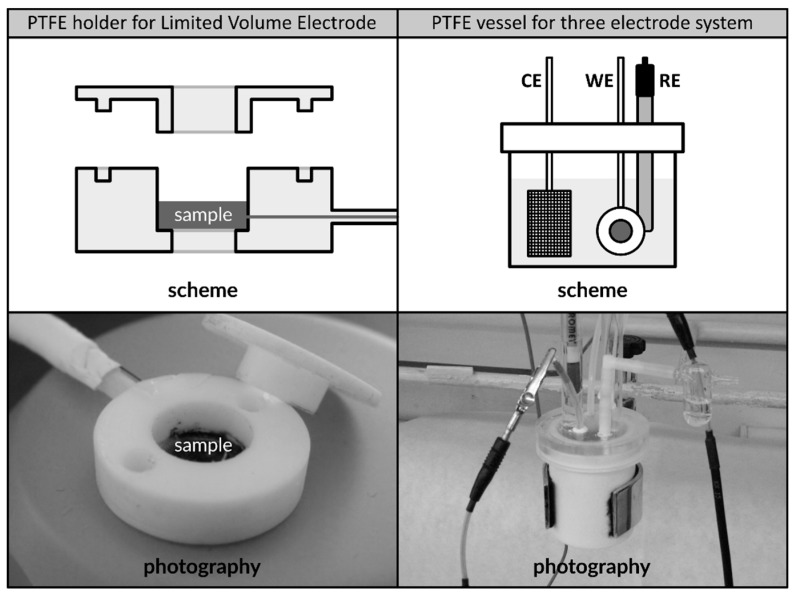
Scheme and photography of PTFE sample holder dedicated to secure limited volume electrodes made of LaMm-Ni_4.1_Al_0.3_Mn_0.4_Co_0.45_ alloy (**left**); PTFE vessel for three-electrode setup including WE—working electrode placed in PTFE holder, RE—reference electrode, and CE—counter electrode (**right**). The schemes are not presented to scale.

The thickness of the electrodes of ca. 50–60 µm reflected the size the alloy’s particles. A dedicated PTFE sample holder was used in all performed experiments to secure the working electrode, which had a diameter of ca. 1 cm, in three electrode systems (Figure 1) [24,25,26,27].

### 2.2. Electrochemical Setup and Techniques

Electrochemical measurements were carried out in the three-electrode setup using AB_5_-LVE (working), Hg|HgO (reference, 6M KOH, HYDROMET, Poland) and gold sheet (counter, Mint of Poland) using 40 mL portions of electrolyte and dedicated 50 mL PTFE vessel. Solutions were purged with argon (5.0, Messer) for 10 min prior to measurement, while overhead space was purged throughout the entire measurement. Potential values presented in this study refer to Hg|HgO reference electrode (−0.944 V vs. RHE at 6M KOH).

Chronoamperometric (CA), chronopotentiometric (CP) and voltammetric (CV) tests were performed using an AUTOLAB 30 analyser (Autolab, Utrecht, The Netherlands). Temperature was stabilised in the range from −30 to +40 °C using Lauda Proline 855 thermostat (Lauda, Lauda-Königshofen, Germany) with KRYO51 bath. Prior to the measurements, the working electrode was activated in a standardised procedure consisting of 50 cycles in a potential range from −1.1 V to −0.4 V, with a 2 mV/s scanning rate, which enabled the electrode to reach capacity value plateau [24]. 

The capacity of the electrode was determined based on the discharge time in galvanostatic measurements with a C-rate of 0.15 C (i.e., full discharge at 1 h/0.15 C = 6.66 h = 400 min). Discharge current values were calculated with reference to the total maximum capacity of the alloy (289 mAh/g) obtained in the PCT tests, i.e., 1 C = 289 mA/g. The capacity of the working electrode at various conditions (in mAh/g) was obtained by multiplication of discharge time (defined in hours) by discharge current (defined in mA/g).

### 2.3. Electrolytes

Aqueous electrolytes were prepared using deionised water purified in a two-step procedure involving distillation and reverse osmosis filtration using Hydrolab (Hydrolab, Straszyn, Poland), followed by UV irradiation using Milipore Simplicity 185 (Millipore, Burlington, MA, US). Alkali metal hydroxides were purchased from POCh (Gliwice, Poland) (LiOH, NaOH, KOH) and Sigma Aldrich (St. Louis, MO, USA) (KOH, RbOH, CsOH) [24,26,27]. 

Three types of electrolytes were used in this study: neat 1M solutions (LiOH, NaOH, KOH, RbOH, CsOH), neat 6M solutions (NaOH, KOH, RbOH, CsOH, note that 6M LiOH was not achievable due to low solubility of LiOH in water), and binary-mixed 6M solutions comprising 4M KOH component and 2M component of another alkali metal hydroxide (LiOH/KOH, NaOH/KOH, RbOH/KOH, CsOH/KOH) [24,26,27]. Results collected for 6M KOH were usually set together with the results of binary 6M electrolytes for easier comparison.

## 3. Results

Previously, we have reported LaMm-Ni_4.1_Al_0.3_Mn_0.4_Co_0.45_ hydrogen storage alloy to be used as negative electrode in Ni-MH batteries [24,26,27]. We have observed that overall performance of the alloy strongly depends on temperature, reaching maximum at 20–30 °C [26] (Figures S9 and S10), which is consistent with earlier reports of Fetcenko and Koch [1] and Gamboa et al. [39]. Also, we have observed strong dependency of the capacity of the alloy on concentration and composition of alkali metal electrolytes [24,26]. The highest capacity, achieving ca. 95% of its maximum theoretical capacity, was observed in 6M KOH (275.1 mAh/g) and in binary mixed 6M LiOH/KOH solution (276.1 mAh/g) [26]. Interestingly, electrode capacity at 6M NaOH/KOH solution, which was far from the maximum value increased at lower temperatures while in all other cases the capacity decreased with decreasing temperature [26]. On the other hand, we observed very low capacities at electrolytes containing RbOH and CsOH [24,26], which was attributed to ongoing corrosion of the alloy. In fact, corrosion of the material was enhanced when in contact with Rb^+^ and Cs^+^ cations, resulting in formation of ternary oxides degrading the electrode and blocking its surface [27] (Figures S11–S19). Thus, instead of gradually increasing degradation of the alloy in a function of strength of the base and an ionic radius of its metal cation, we observed jump of corrosion intensity at RbOH and CsOH.

### 3.1. Low Temperature Performance of the Alloy in 6M Single Cation Electrolytes

**Figure 2:**
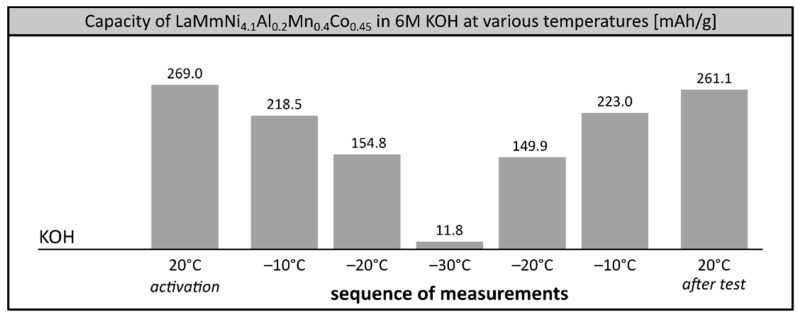
Electrochemical capacity of LaMm–Ni_4.1_Al_0.3_Mn_0.4_Co_0.45_ alloy at 6M KOH electrolyte at low temperatures. Sequence of temperature changes: +20 °C (activation), −10 °C, −20 °C, −30 °C, −20 °C, −10 °C, +20 °C (reference measurement).

In our previous study, we showed that the maximum performance of the investigated AB_5_ alloy among single cation 6M electrolytes could be observed in KOH solution over the entire investigated temperature range 0–30 °C [22], which is in good agreement with other reports [40]. Thus, we attempted to check the performance and stability of the alloy soaked in 6M KOH and other 6M electrolytes at extremely low temperatures, reaching −30 °C. 1M electrolytes were not investigated in this temperature range due to their high freezing point. Surprisingly, in 6M KOH we observed poor performance of the electrode at low temperature (Figure 2.). At −20 °C the capacity of the alloy was only 56.3% (154.8 mAh/g) of the initial value and further dropped down to mere 4.3% (11.8 mAh/g) at −30 °C, which was comparable with commercial Ni-MH systems [8,9]. Apparently, 6M KOH solution, considered to be the most appropriate solvent for Ni-MH batteries under ambient conditions [1,41], balancing positive aspect of high ionic conductivity of concentrated electrolyte with relatively low degradation of an alloy, does not behave optimally in low temperature ranges. However, the stability of the investigated alloy remained very good, with the full electrochemical capacity of the alloy recovered in a reference measurement when the system was reheated to room temperature after the low temperature tests.

**Figure 3:**
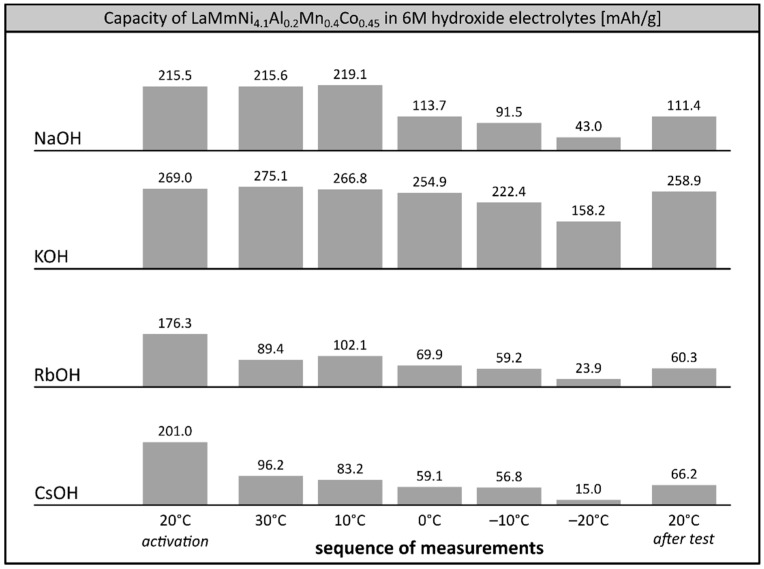
Electrochemical capacity of LaMm–Ni_4.1_Al_0.3_Mn_0.4_Co_0.45_ alloy at 6M alkali metal hydroxide electrolytes (NaOH, KOH, RbOH, CsOH) at decreasing temperatures. Sequence of temperature changes: +20 °C (activation), +30 °C, +10 °C, 0 °C, −10 °C, −20 °C, +20 °C (reference measurement).

Performance of LaMm-Ni_4.1_Al_0.3_Mn_0.4_Co_0.45_ alloy in other single cation 6M electrolytes appeared much worse than in 6M KOH and very far from the parameters characteristic for commercial Ni-MH systems [7] (Figure 3). After activation at 20 °C, the capacity of the alloy significantly decreased in each investigated electrolyte, resulting in very poor values observed at −20 °C. In 6M NaOH the alloy maintained only 16.5% (43.0 mAh/g) of its initial capacity, while in solutions of heavier alkali metals, the observed capacity was even lower (23.9 mAh/g at 6M RbOH, 15.0 mAh/g at 6M CsOH). Also, the electrode material did not reach its starting capacity in the reference measurement back at room temperature, suggesting irreversible degradation of the alloy, relatively mild at 6M NaOH, and significant at RbOH and CsOH, probably due to ongoing very intensive corrosion processes, as reported earlier [27].

### 3.2. Low Temperature Performacne of the Alloy in 6M Binary Mixed Electrolytes

**Figure 4:**
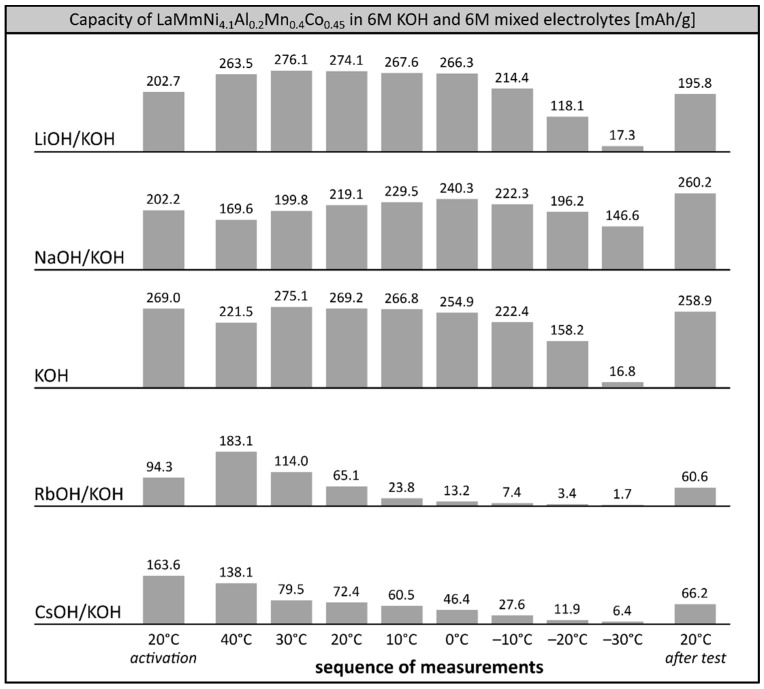
Electrochemical capacity of LaMm–Ni_4.1_Al_0.3_Mn_0.4_Co_0.45_ alloy at 6M KOH electrolyte and 6M mixed alkali metal hydroxide electrolytes (LiOH/KOH, NaOH/KOH, RbOH/KOH, CsOH/KOH) at decreasing temperatures. Sequence of temperature changes: +20 °C (activation), +40 °C, +30 °C, +20 °C, +10 °C, 0 °C, −10 °C, −20 °C, −30 °C, +20 °C (reference measurement).

Bearing in mind that the overall performance and stability of LaMm-Ni_4.1_Al_0.3_Mn_0.4_Co_0.45_ alloy can be significantly improved by optimisation of the composition of the electrolyte [26], we performed extended high- and low-temperature tests of the working electrode at binary mixed 6M solutions based on KOH (Figure 4). Tests of the alloy at pristine 6M KOH were performed as a reference system. Apparently, high temperature treatment (+40 °C) prior to low temperature study slightly enhances the capacity of the alloy at −30 °C, reaching 6.1% (16.8 mAh/g) of its maximum capacity. As in previous sequences of measurements, the stability of the alloy in 6M KOH is very good, retaining over 96% of its starting capacity.

The properties of the alloy at 6M LiOH/KOH electrolyte were slightly better (1–5%) than in 6M KOH above 0 °C, but at negative temperatures, the capacity of the material dropped quicker than in KOH, stopping at a very similar value (17.3 mAh/g) at −30 °C.

The behaviour of the investigated alloy soaked in 6M mixed electrolytes containing rubidium and caesium cations (RbOH/KOH, CsOH/KOH) was very poor. In both electrolytes, the initial capacity was relatively low, due to ongoing corrosion processes, and dropped further to negligible values already at temperatures below −10 °C.

Surprisingly, capacity changes of the studied alloy soaked in binary 6M NaOH/KOH electrolyte were of a different nature to those in pristine KOH and binary LiOH/KOH electrolytes. After slight initial decrease at +40 °C (169.6 mAh/g), the capacity increased with decreasing temperature to reach a maximum value of 240.3 mAh/g at 0 °C. Further cooling to negative temperatures resulted in a slow decrease of the capacity to the values of 222.3 mAh/g at −10 °C, 196.2 mAh/g at −20 °C and 146.6 mAh/g at −30 °C, equal to 92.5%, 81.7% and 61.0% of the maximum capacity recorded at 0 °C. The observed preservation of the electrochemical capacity of the alloy was significantly higher than in 6M KOH and in commercially available Ni-MH systems, already able to retain less than 60% of their initial capacity at −20 °C [8,9]. It is worth mentioning that the capacity of the investigated alloy increased above the initial capacity when reheated to room temperature. This may suggest desorption of some additional portions of hydrogen trapped at negative temperatures; this was not observed, however, in the other electrolytes. It is more likely that the alloy soaked in 6M NaOH/KOH underwent a non-standard low temperature activation processes, which enhanced its low temperature and high temperature performance parameters.

### 3.3. Hydrogen Diffusion Coefficient in Alloy Alkali Metal Hydroxide Electrolytes

**Figure 5:**
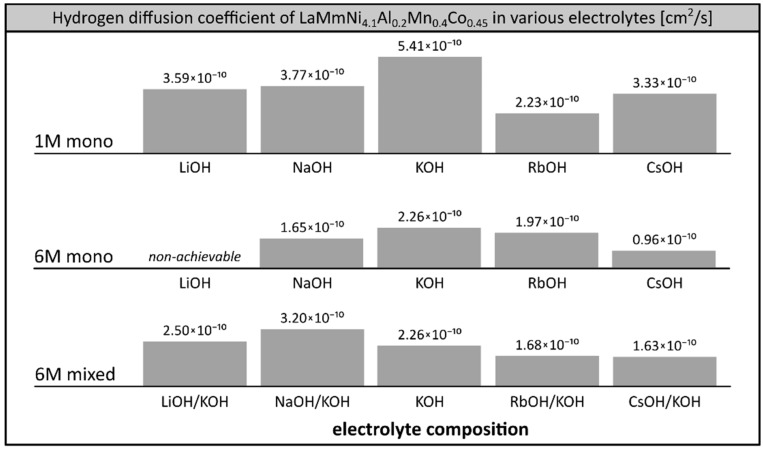
Hydrogen diffusion coefficient in LaMm–Ni_4.1_Al_0.3_Mn_0.4_Co_0.45_ alloy as a function of a composition of 1M alkali metal electrolytes (LiOH, NaOH, KOH, RbOH, CsOH), 6M alkali metal electrolytes (NaOH, KOH, RbOH, CsOH) and binary mixed 6M alkali metal hydroxide electrolytes (LiOH/KOH, NaOH/KOH, RbOH/KOH, CsOH/KOH), determined in chronopotentiometric tests. Temperature 20 °C, SOC = 1.

Hydrogen diffusion coefficients were determined at room temperature in all the electrolytes tested previously (Figure 5) using a procedure described before [24,26]. Higher values of hydrogen diffusion coefficient observed in 1M electrolytes (e.g., 5.41 × 10^−10^ cm^2^/s for 1M KOH) in comparison to 6M solutions (e.g., 2.26 × 10^−10^ cm^2^/s for 6M KOH) are in agreement with earlier reports [42]. This can be explained by the lower electrode loading levels (state of charge, SOC) achievable at diluted electrolytes, which result in the formation of hydrogen solution in solid metal (low SOC, phase α) where hydrogen is free to migrate, in contrast to metal hydride (high SOC, phase β) where mobility of hydrogen is inhibited. Thus, lower values of hydrogen diffusion coefficients were observed in better performing systems using 6M electrolytes, which is generally to be expected. The determined values of hydrogen diffusion coefficient at SOC = 1 (average ca. 2.5 × 10^−10^ cm^2^/s) lay within the range observed for other hydrogen storage alloys of similar grain size [43,44,45].

In the systems investigated here, we observed the well-known correlation of determined hydrogen diffusion coefficient with the performance of the working electrode (Figure 5). The maximum capacity was recorded in the systems exhibiting the highest hydrogen diffusion rate among the systems using the same concentration of electrolyte. In the case of single cation electrolytes, in both series of 1M and 6M solutions, the highest capacities and the largest values of hydrogen diffusion coefficients were observed for KOH systems. The pronounced correlation was also observed in the case of binary mixed 6M electrolytes, where the hydrogen diffusion coefficient was the highest in the best performing systems: NaOH/KOH (3.20 × 10^−10^ cm^2^/s), which had a capacity superior to KOH in the low temperature region; and LiOH/KOH (2.50 × 10^−10^ cm^2^/s), which was superior to KOH at room temperature.

## 4. Discussion

Inferior performance of Ni-MH batteries at low temperatures in comparison to ambient conditions can be explained by multiple factors, worsening the hydrogen storage properties of the alloy forming negative electrodes, most of which relate to electrolyte characteristics and its interaction with the electrode. The deteriorating trend of properties of MH electrodes is also manifested in the decreasing hydrogen diffusion coefficients at low temperatures [40], which are known to reflect the overall capacity of the electrode. 

One of the key factors in the overall performance of the system is the conductivity of the electrolyte, which needs to be high enough to allow efficient charge transfer, which explains the superior capacities obtained with 6M electrolytes in comparison to 1M systems. Conductivity of the electrolytes decreases by ca. an order of magnitude with decreasing temperature from 20 to −30 °C [7,40]. Also, other meaningful parameters change with decreasing temperature (e.g., charge transfer resistance and polarisation resistance grow significantly), further inhibiting the electrode reaction rate [40]. Interestingly, KOH has the highest conductivity among the alkali metal hydroxide aqueous solutions, reaching its maximum value at a concentration of ca. 6M. LiOH and NaOH solutions exhibit lower conductivity than KOH because Li^+^ and Na^+^, due to their smaller ionic radius, appear to be coordinated by water molecules, impeding their mobility. On the other hand, RbOH and CsOH solutions also show lower conductivity than KOH, because the larger ionic radii of Rb^+^ and Cs^+^ result in lower charge density of these ions, impeding their mobility in the electric field. Thus, any doping of KOH solution with other alkali metal hydroxide results in a drop in overall conductivity of the system by a few percent [46]. This phenomenon would suggest that the highest performance is to be expected in pristine 6M KOH. Meanwhile, we observed superior performance of the AB_5_ electrode at 6M NaOH/KOH at low temperature and in 6M LiOH/KOH under ambient conditions, in comparison to 6M KOH. This suggests that other factors overcompensate the effect of the relatively poor conductivity of the mixed electrolytes.

Another key factor at low temperatures is the increasing viscosity of the electrolytes, especially when the temperature is only slightly above freezing point. The viscosity of KOH water solution is known to grow by ca. an order of magnitude when the temperature drops from 0 to −30 °C [47], and similar behaviour is expected for other alkali metal hydroxide solutions. At room temperature, the dynamic viscosity of KOH is lower than the viscosity of NaOH, and this difference cannot be compensated by differences in the density of these solvents [48]. Thus, the viscosity of the mixed electrolyte should not be lower than the viscosity of neat KOH solutions. This suggests that other factors need overcompensate the effect increased viscosity of mixed electrolytes. 

The above-mentioned solvent effects suggest that corrosion effects might play the key role in the enhancement of the low temperature properties of LaMm–Ni_4.1_Al_0.3_Mn_0.4_Co_0.45_ alloy. The selective oxidation characteristics of AB_5_ materials soaked in concentrated hydroxide solutions is known to diminish the overall parameters of the MH electrodes [18,20,21,22]. The intensity of degradation of the alloy is correlated with the strength of the hydroxide used, and thus with the ionic radius of the constituent metal cation [27]. However, the corrosion effects are significantly enhanced when metal cations from the solution can react with the alloy to form additional corrosion products, which is the case of rubidium- and caesium-containing solutions, where numerous ternary oxides can be formed [27]. 

The intensity of corrosion observed in 6M NaOH/KOH electrolyte was significantly lower in comparison to other electrolytes, including 6M KOH [27], which could explain the enhanced low temperature capacity of the alloy in this solution. A similar effect in 6M LiOH/KOH was not expected, since LiOH is less soluble in water, and thus forms more viscous solutions. In general, LiOH additive is believed to mainly enhance durability and high temperature capacity of hydrogen storage alloys [1].

## 5. Summary and Conclusions

We presented a detailed study of the low temperature properties of hydrogen storage metal alloy LaMm-Ni_4.1_Al_0.3_Mn_0.4_Co_0.45_ (AB_5_-type) in 6M alkali metal hydroxide solutions of various composition (LiOH, NaOH, KOH, RbOH, CsOH, LiOH/KOH, NaOH/KOH, RbOH/KOH, CsOH/KOH). We observed a dramatic decrease of electrochemical capacity of the alloy below −20 °C in most of the investigated electrolytes, including the best-performing 6M KOH. Surprisingly, the working electrode soaked in 6M NaOH/KOH was able to maintain as much as 81.7% (196.2 mAh/h) and 61.0% (146.6 mAh/h) of its maximum capacity (240.3 mAh/g) at −20 °C and −30 °C, respectively, and further regain its starting capacity after reheating to room temperature. The observed preservation of the electrode’s capacity in the latter system is superior to the commercially available configurations, which can maintain less than 60% of their nominal capacity already at −20 °C [8,9].

We observed a clear correlation of the electrode’s capacity with the measured hydrogen diffusion coefficient. Among the 6M electrolytes, the maximum hydrogen diffusion coefficient was determined in NaOH/KOH (3.20 × 10^−10^ cm^2^/s), exceeding that of other well-performing solutions: LiOH/KOH (2.50 × 10^−10^ cm^2^/s) and KOH (2.26 × 10^−10^ cm^2^/s).

Enhanced low temperature properties of the working electrode soaked in 6M NaOH/KOH electrolyte may emerge from the possible effect of inhibited surface corrosion, which appear to be more significant than the expected negative effects of lowering conductivity and increased viscosity of the electrolyte resulting from doping of KOH with Na^+^ cations. 

The presented study may be important for designing novel electrolytes that are less viscous and more conductive to be used in Ni-MH batteries, suggesting preparation of NaOH containing ternary or quaternary electrolytes. However, further investigation regarding the role of sodium cations and their synergy with potassium cations is still needed.

## Supplementary Materials

The following are available online at https://www.mdpi.com/1996-1944/12/24/4220/s1, Figure S1: SEM images of LaMmNi_4.1_Al_0.2_Mn_0.4_Co_0.45_ alloy particles (top), group of particles (middle) and limited volume electrode formed by compression of the alloy between two sheets of gold mesh (bottom), Figure S2: Comparison of X-ray powder diffractograms of LaMmNi_4.1_Al_0.2_Mn_0.4_Co_0.45_ alloy (λ = 1.78901 Å) [24], Figure S3: Unit cell of LaMmNi_4.1_Al_0.2_Mn_0.4_Co_0.45_ alloy, a = b = 5.0079(5) Å, c = 4.0521(4) Å, V = 88.007(16) Å^3^ [24], Figure S4: EDS images of the surface of pristine LaMmNi_4.1_Al_0.2_Mn_0.4_Co_0.45_ alloy with mapping of elemental distribution of lanthanum, nickel, cerium, cobalt, manganese and aluminium [27], Figure S5: EDS spectrum of the surface of pristine LaMmNi_4.1_Al_0.2_Mn_0.4_Co_0.45_ alloy not subjected to electrochemical treatment [24], Figure S6: Distribution of the particle size of the LaMmNi_4.1_Al_0.2_Mn_0.4_Co_0.45_ alloy: pristine alloy (bottom), hydrogenated alloy (middle) and alloy after 5 cycles of hydrogenation with gaseous H_2_ (top). Particle size [μm] showed in a function of volume fraction Bv [%]. Percentile values of 10%, 50% and 90% of the population exposed with grey fields. Sample population 200,000 particles. [24,27], Figure S7: Comparison of hydrogen absorption and desorption isotherms at temperatures below 60 °C of: LaMmNi_4.1_Al_0.2_Mn_0.4_Co_0.45_ alloy (left) [26], and pristine LaNi_5_ alloy [17], Figure S8: Comparison of hydrogen absorption isotherms at temperature range 30–150 °C of: LaMmNi_4.1_Al_0.2_Mn_0.4_Co_0.45_ alloy (left) [26], and pristine LaNi_5_ alloy [17], Figure S9: Electrochemical capacity of LaMm–Ni_4.1_Al_0.3_Mn_0.4_Co_0.45_ alloy as a function of composition of 1M and 6M MOH (M = Li, Na, K, Rb, Cs) solutions at temperatures in the range 0–30 °C in a series of decreasing temperatures [26], Figure S10: Electrochemical capacity of LaMm–Ni_4.1_Al_0.3_Mn_0.4_Co_0.45_ alloy as a function of composition of 6M MOH/KOH (M = Li–Cs) solutions at temperatures in the rang 0–30 °C in a series of decreasing temperature [26], Figure S11: EDS images of LaMmNi_4.1_Al_0.2_Mn_0.4_Co_0.45_ alloy electrochemically treated in 1M LiOH at 30 °C [27], Figure S12: EDS images of LaMmNi_4.1_Al_0.2_Mn_0.4_Co_0.45_ alloy electrochemically treated in 6M LiOH/KOH at 30 °C [27], Figure S13: EDS images of LaMmNi_4.1_Al_0.2_Mn_0.4_Co_0.45_ alloy electrochemically treated in 6M NaOH at 30 °C [27], Figure S14: EDS images of LaMmNi_4.1_Al_0.2_Mn_0.4_Co_0.45_ alloy electrochemically treated in 6M NaOH/KOH at 30 °C [27], Figure S15: EDS images of LaMmNi_4.1_Al_0.2_Mn_0.4_Co_0.45_ alloy electrochemically treated in 6M KOH at 30 °C [27], Figure S16: EDS images of LaMmNi_4.1_Al_0.2_Mn_0.4_Co_0.45_ alloy electrochemically treated in 6M RbOH at 30 °C [27], Figure S17: EDS images of LaMmNi_4.1_Al_0.2_Mn_0.4_Co_0.45_ alloy electrochemically treated in 6M RbOH/KOH at 30 °C [27], Figure S18: EDS images of LaMmNi_4.1_Al_0.2_Mn_0.4_Co_0.45_ alloy electrochemically treated in 6M CsOH at 30 °C [27], Figure S19: EDS images of LaMmNi_4.1_Al_0.2_Mn_0.4_Co_0.45_ alloy electrochemically treated in 6M CsOH/KOH at 30 °C [27], Table S1: Occupation of crystallographic sites in the crystal structure of LaMmNi_4.1_Al_0.2_Mn_0.4_Co_0.45_ alloy [24], Table S2: Results of EDS elemental analysis of pristine LaMmNi_4.1_Al_0.2_Mn_0.4_Co_0.45_ alloy not subjected to electrochemical treatment [24], Table S3: Results of granulometric determination of particle size distribution of the LaMmNi_4.1_Al_0.2_Mn_0.4_Co_0.45_ alloy: pristine alloy, hydrogenated alloy and alloy after 5 cycles of hydrogenation with gaseous H_2_. [24,27].
