# Supplementary material for: Low Temperature Characteristics of Hydrogen Storage Alloy LaMm-Ni4.1Al0.3Mn0.4Co0.45 for Ni-MH Batteries
Source: Materials (Basel). 2019 Dec 16;12(24):4220. doi: 10.3390/ma12244220 (PMC6947314; doi:10.3390/ma12244220)
Supplement: Supplementary file 1 [file materials-12-04220-s001.pdf]

materials

MDPI

Supplementary Materials

Low Temperature Characteristics of Hydrogen Storage Alloy LaMm-$Ni_{4.1}Al_{0.3}Mn_{0.4}Co_{0.45}$ for Ni-MH Batteries

Malgorzata Karwowska, Karol J. Fijalkowski and Andrzej A. Czerwiński

Table of Contents:

1. LaMm-$Ni_{4.1}Al_{0.3}Mn_{0.4}Co_{0.45}$ alloy

– SEM images of the alloy particles and limited volume electrode
– Powder X-ray diffraction patterns of the alloy
– View of the unit cell
– Occupation of crystallographic sites
– EDS images of the surface of the alloy
– EDS spectrum and numeric results of EDS analysis of the surface of the alloy

2. Sorption of gaseous hydrogen by LaMm-$Ni_{4.1}Al_{0.3}Mn_{0.4}Co_{0.45}$ alloy

– Particle size distribution spectra of the alloy upon sorption of gaseous hydrogen
– Particle size distribution data of the alloy upon sorption of gaseous hydrogen
– Hydrogen absorption and desorption isotherms at ambient conditions
– Hydrogen absorption isotherms in temperature range of 30–150°C

3. Electrochemical capacity of LaMm-$Ni_{4.1}Al_{0.3}Mn_{0.4}Co_{0.45}$ alloy in alkaline solutions

– Electrochemical capacity of the alloy in 1M and 6M MOH solutions
– Electrochemical capacity of the alloy in 6M MOH/KOH solutions

4. SEM images of corrosion structures at the surface of LaMm-$Ni_{4.1}Al_{0.3}Mn_{0.4}Co_{0.45}$ alloy

– Corrosion formations after soaking in LiOH solutions
– Corrosion formations after soaking in LiOH/KOH solutions
– Corrosion formations after soaking in NaOH solutions
– Corrosion formations after soaking in NaOH/KOH solutions
– Corrosion formations after soaking in KOH solutions
– Corrosion formations after soaking in RbOH solutions
– Corrosion formations after soaking in RbOH/KOH solutions
– Corrosion formations after soaking in CsOH solutions
– Corrosion formations after soaking in CsOH/KOH solutions

1. LaMm-$Ni_{4.1}Al_{0.3}Mn_{0.4}Co_{0.45}$ alloy [1/4]

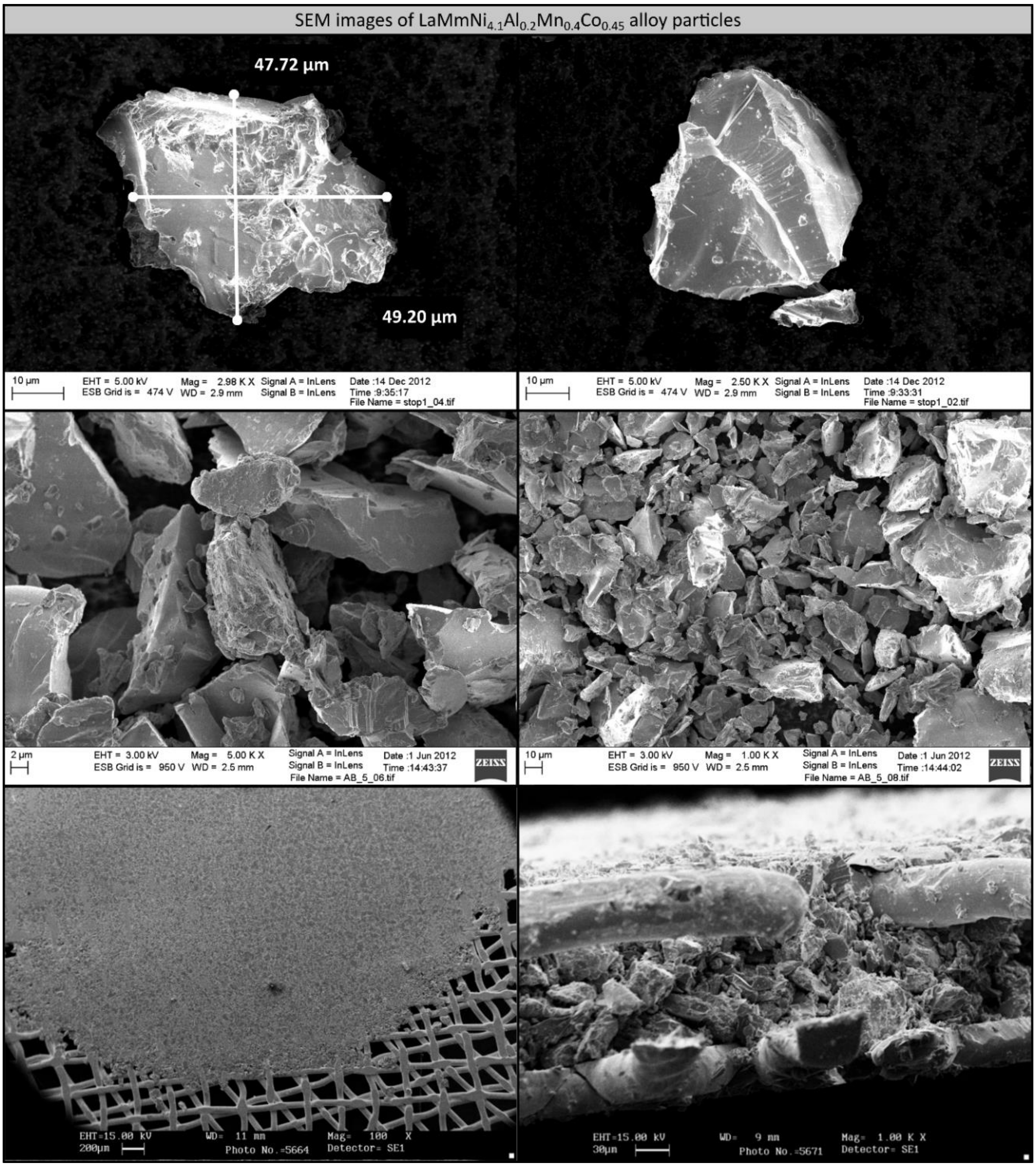

Figure S1. SEM images of $LaMmNi_{4.1}Al_{0.2}Mn_{0.4}Co_{0.45}$ alloy particles (top), group of particles (middle) and limited volume electrode formed by compression of the alloy between two sheets of gold mesh (bottom).

1. LaMm-$Ni_{4.1}Al_{0.3}Mn_{0.4}Co_{0.45}$ alloy [2/4]

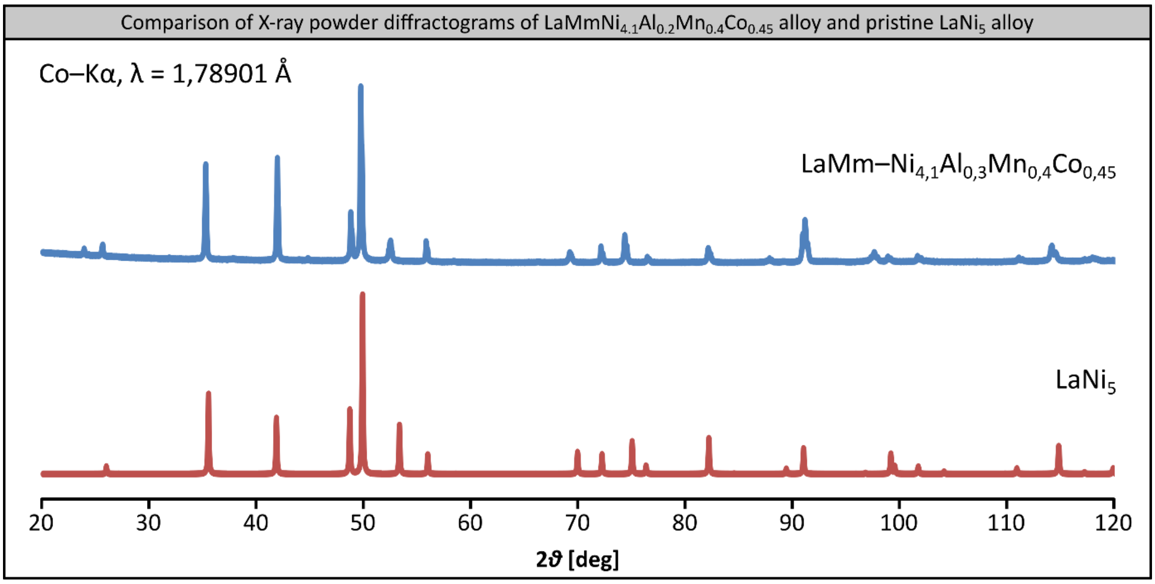

Figure S2. Comparison of X-ray powder diffractograms of LaMm$Ni_{4.1}Al_{0.2}Mn_{0.4}Co_{0.45}$ alloy (λ = 1.78901 Å) [M. Karwowska et al., J. Power Sources, 263 (2014) 304] $LaNi_5$ alloy [H.N. Nowotny, Z. Metallkd. 34 (1942) 247].

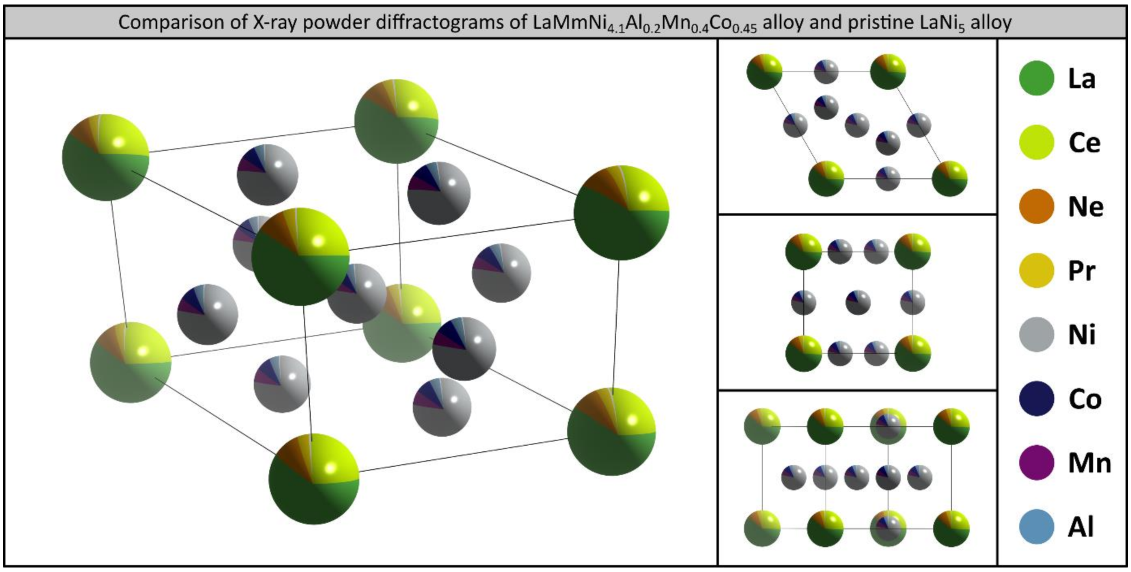

Figure S3. Unit cell of LaMm$Ni_{4.1}Al_{0.2}Mn_{0.4}Co_{0.45}$ alloy, a = b = 5.0079(5) Å, c = 4.0521(4) Å, V = 88.007(16) $Å^3$, [M. Karwowska et al., J. Power Sources, 263 (2014) 304].

Table S1. Occupation of crystallographic sites in the crystal structure of LaMm$Ni_{4.1}Al_{0.2}Mn_{0.4}Co_{0.45}$ alloy [M. Karwowska et al., J. Power Sources, 263 (2014) 304].

Atom	Position	Occupation	Atom	Position 1	Position 2	Occupation
La	0 0 0	60%	Ni	0.67 0.33 0	0.5 0 0.5	78.00%
Ce	0 0 0	26%	Co	0.67 0.33 0	0.5 0 0.5	8.66%
Nd	0 0 0	10%	Mn	0.67 0.33 0	0.5 0 0.5	7.44%
Pr	0 0 0	4%	Al	0.67 0.33 0	0.5 0 0.5	5.90%

1. LaMm-$Ni_{4.1}Al_{0.3}Mn_{0.4}Co_{0.45}$ alloy [3/4]

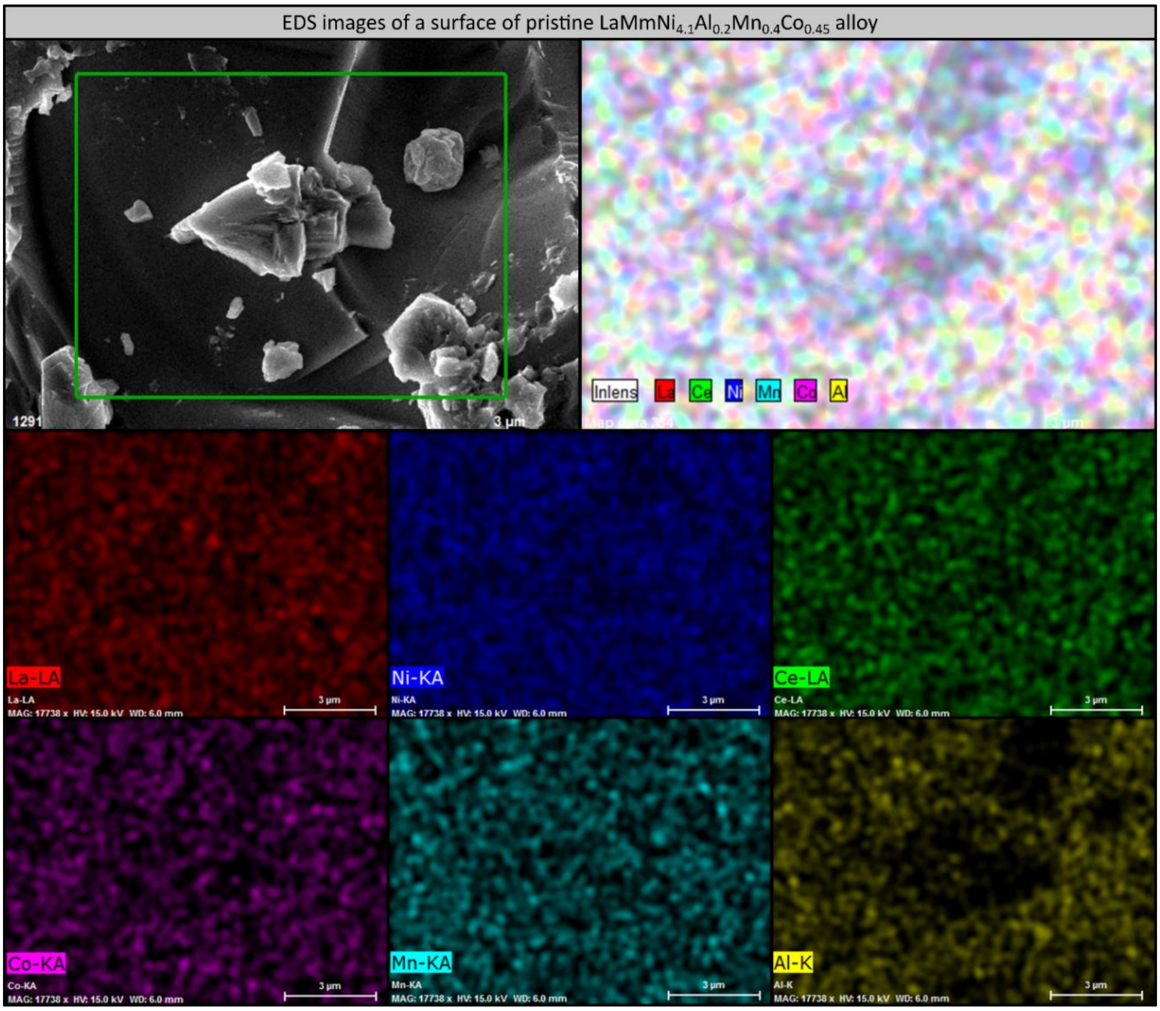

Figure S4. EDS images of the surface of pristine $LaMmNi_{4.1}Al_{0.2}Mn_{0.4}Co_{0.45}$ alloy with mapping of elemental distribution of lanthanum, nickel, cerium, cobalt, manganese and aluminium. [M. Karwowska, et al., Materials., 11 (2018) 2423].

1. LaMm-$Ni_{4.1}Al_{0.3}Mn_{0.4}Co_{0.45}$ alloy [4/4]

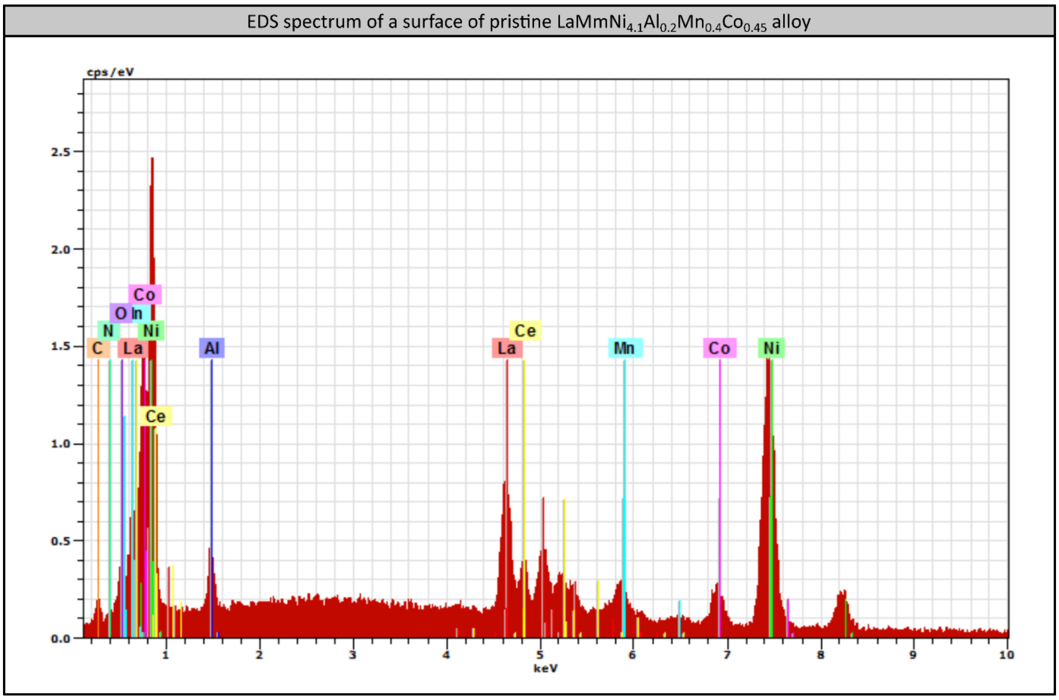

Figure S5. EDS spectrum of the surface of pristine $LaMmNi_{4.1}Al_{0.2}Mn_{0.4}Co_{0.45}$ alloy not subjected to electrochemical treatment [M. Karwowska et al., J. Power Sources, 263 (2014) 304].

Table S2. Results of EDS elemental analysis of pristine $LaMmNi_{4.1}Al_{0.2}Mn_{0.4}Co_{0.45}$ alloy not subjected to electrochemical treatment [M. Karwowska et al., J. Power Sources, 263 (2014) 304].

**Element**	**Series**	**unn. C [wt %]**	**norm. C [wt %]**	**Atom. C [at %]**	**Error [wt %]**
Lanthanum	L-series	19.48	19.91	8.52	0.60
Nickel	K-series	55.72	56.94	57.67	1.77
Aluminium	K-series	1.95	2.00	4.40	0.13
Manganese	K-series	3.99	4.08	4.41	0.16
Cobalt	K-series	5.62	5.75	5.80	0.23
Cerium	L-series	7.63	7.80	3.31	0.26
Carbon	K-series	2.01	2.06	10.19	0.48
Nitrogen	M-series	0.48	0.49	2.07	0.19
Oxygen	K-series	0.96	0.98	3.63	0.23
Total		97.85	100.00	100.00	

2. Sorption of gaseous hydrogen by LaMm-$Ni_{4.1}Al_{0.3}Mn_{0.4}Co_{0.45}$ alloy [1/2]

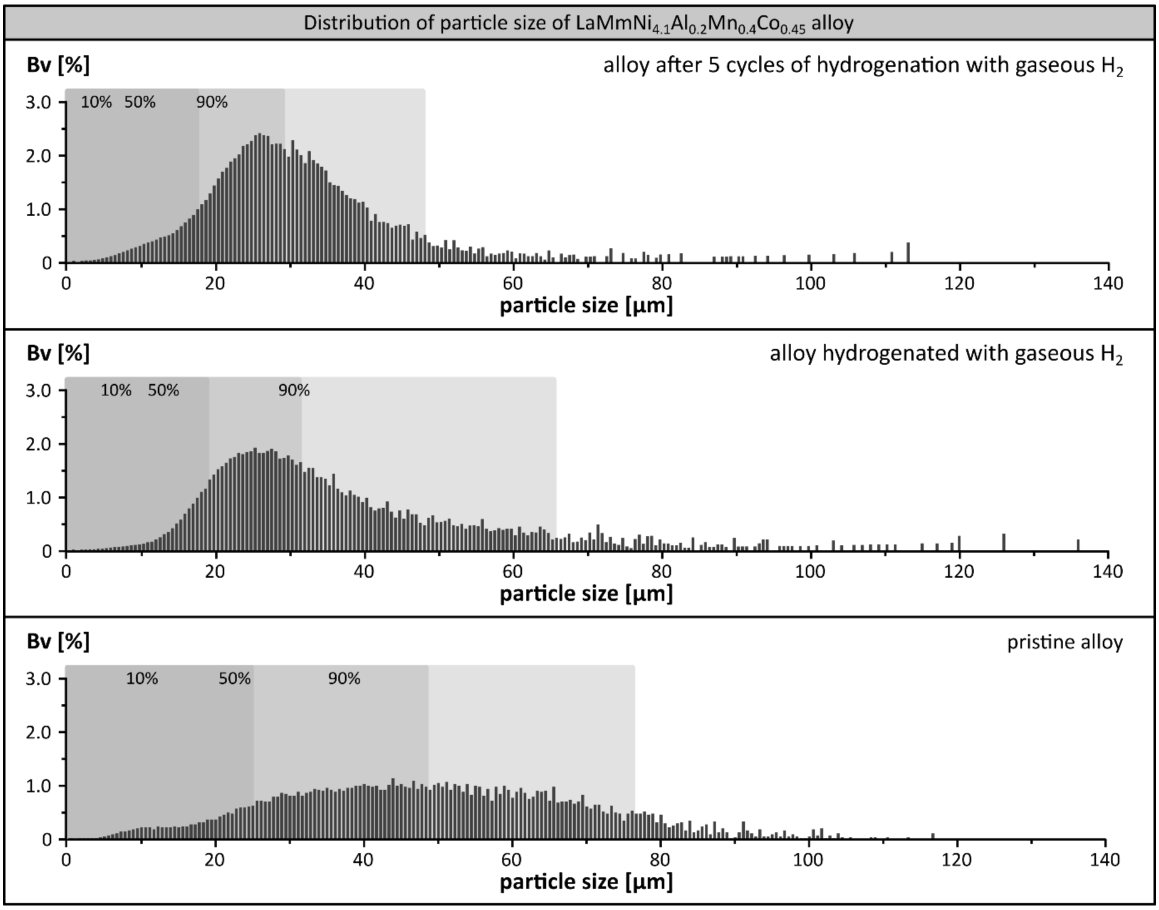

Figure S6. Distribution of the particle size of the $LaMmNi_{4.1}Al_{0.2}Mn_{0.4}Co_{0.45}$ alloy: pristine alloy (bottom), hydrogenated alloy (middle) and alloy after 5 cycles of hydrogenation with gaseous H_2 (top). Particle size [µm] showed in a function of volume fraction Bv [%]. Percentile values of 10%, 50% and 90% of the population exposed with grey fields. Sample population 200,000 particles. [M. Karwowska et al., J. Power Sources, 263 (2014) 304; M. Karwowska et al., Materials, 11 (2018) 2423].

Table S3. Results of granulometric determination of particle size distribution of the $LaMmNi_{4.1}Al_{0.2}Mn_{0.4}Co_{0.45}$ alloy: pristine alloy, hydrogenated alloy and alloy after 5 cycles of hydrogenation with gaseous H_2. Parameters of pristine alloy were shown by us before in [M. Karwowska et al., J. Power Sources, 263 (2014) 304; M. Karwowska et al., Materials, 11 (2018) 2423].

Parameter	Pristine Alloy	Hydrogenated Alloy	Alloy after 5 Cycles
Percentie 10%	24.1 µm	19.3 µm	18.0 µm
Percentie 50%	48.8 µm	32.4 µm	29.5 µm
Percentie 90%	76.7 µm	65.6 µm	47.8 µm
spherical coefficient	1.592	2.091	2.327
Specific mass surface	606 cm^2/g	772 cm^2/g	887 cm^2/g
Specific volume surface	1539 cm^2/cm^3	1961 cm^2/cm^3	2227 cm^2/cm^3

2. Sorption of gaseous hydrogen by LaMm-$Ni_{4.1}Al_{0.3}Mn_{0.4}Co_{0.45}$ alloy [2/2]

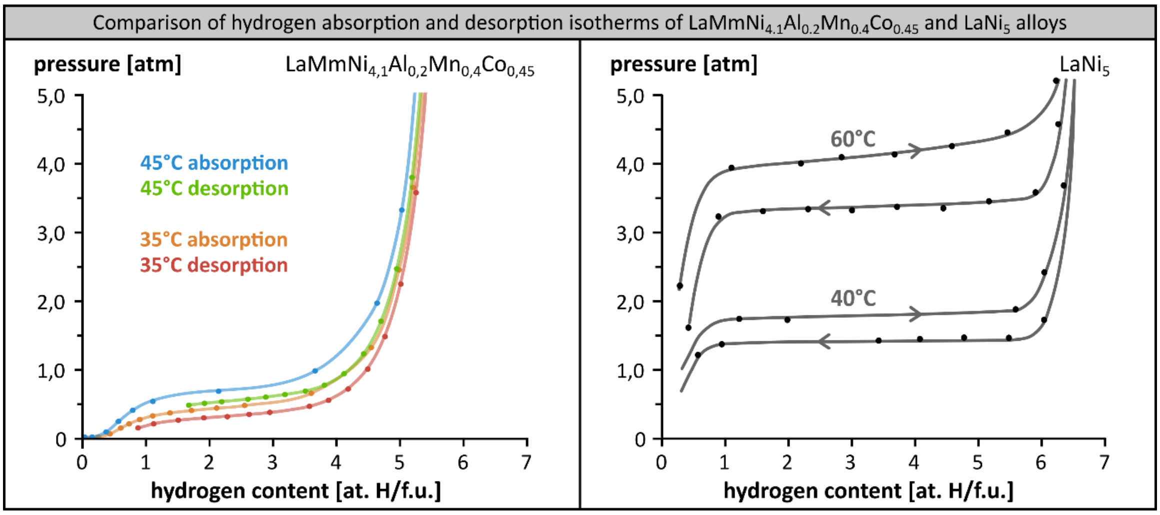

Figure S7. Comparison of hydrogen absorption and desorption isotherms at temperatures below 60 °C of: $LaMmNi_{4.1}Al_{0.2}Mn_{0.4}Co_{0.45}$ alloy (left) [M. Karwowska et al., J. Power Sources, 263 (2014) 304], and pristine $LaNi_5$ alloy [H. H. van Mal, PhD Thesis, Technische Hogenschool, Delft 1976].

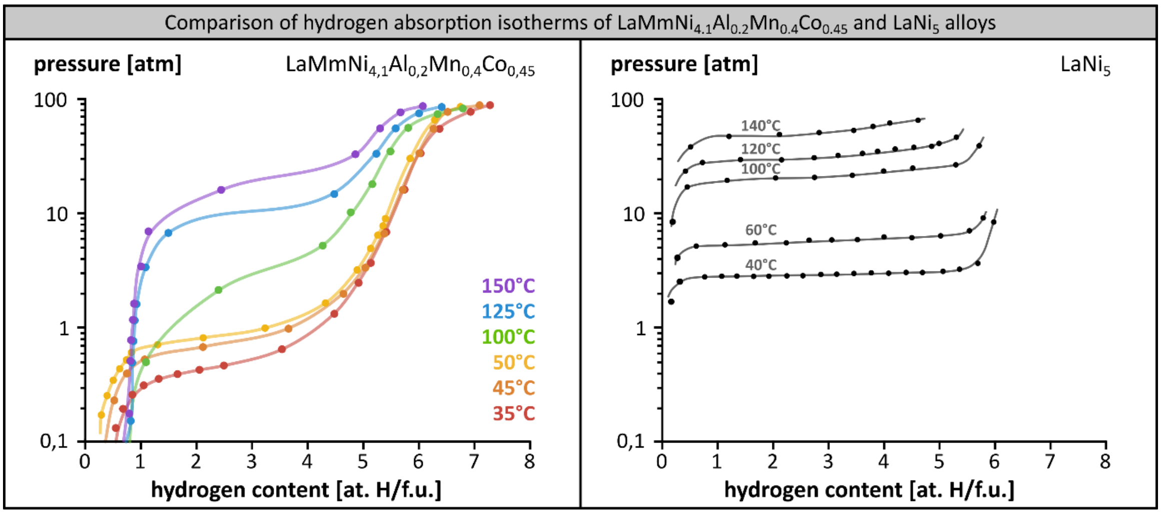

Figure S8. Comparison of hydrogen absorption isotherms at temperature range 30–150 °C of: $LaMmNi_{4.1}Al_{0.2}Mn_{0.4}Co_{0.45}$ alloy (left) [M. Karwowska et al., J. Power Sources, 263 (2014) 304], and pristine $LaNi_5$ alloy [H. H. van Mal, PhD Thesis, Technische Hogenschool, Delft 1976].

3. Electrochemical capacity of LaMm-$Ni_{4.1}Al_{0.3}Mn_{0.4}Co_{0.45}$ alloy in alkaline solutions

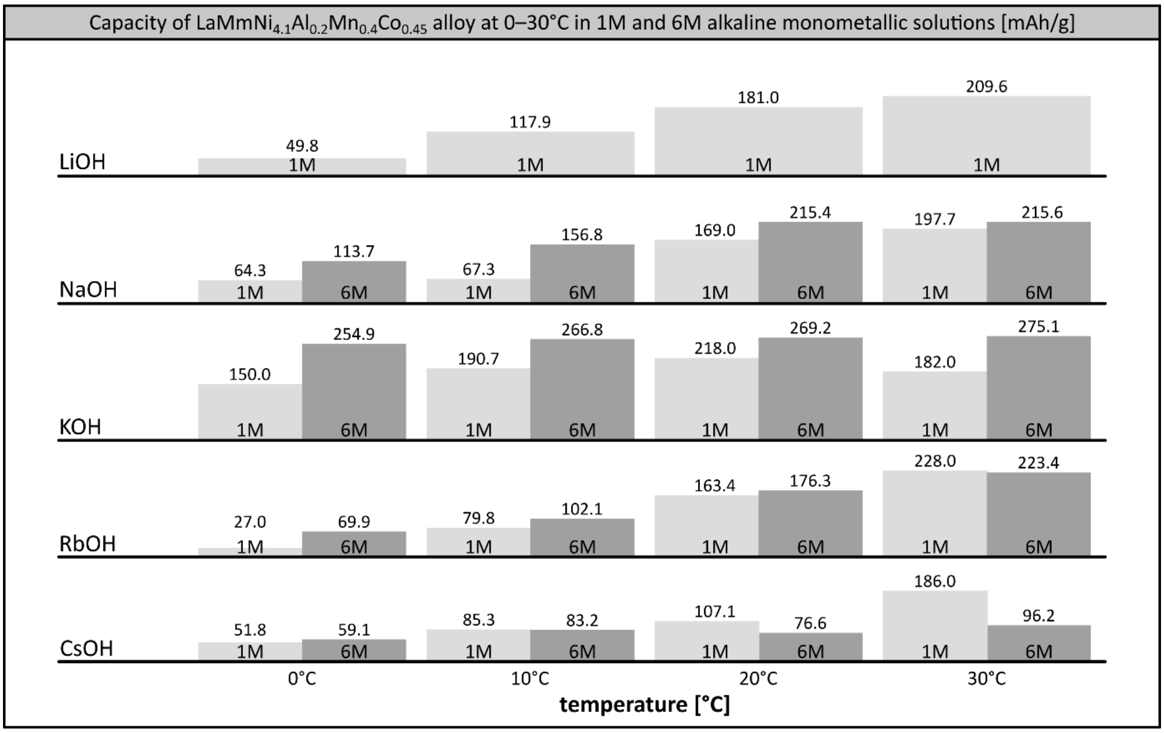

Figure S9. Electrochemical capacity of LaMm–$Ni_{4.1}Al_{0.3}Mn_{0.4}Co_{0.45}$ alloy as a function of composition of 1M and 6M MOH (M = Li, Na, K, Rb, Cs) solutions at temperatures in the range 0–30 °C in a series of decreasing temperatures [M. Karwowska et al., Electrochim. Acta., 252 (2017) 381].

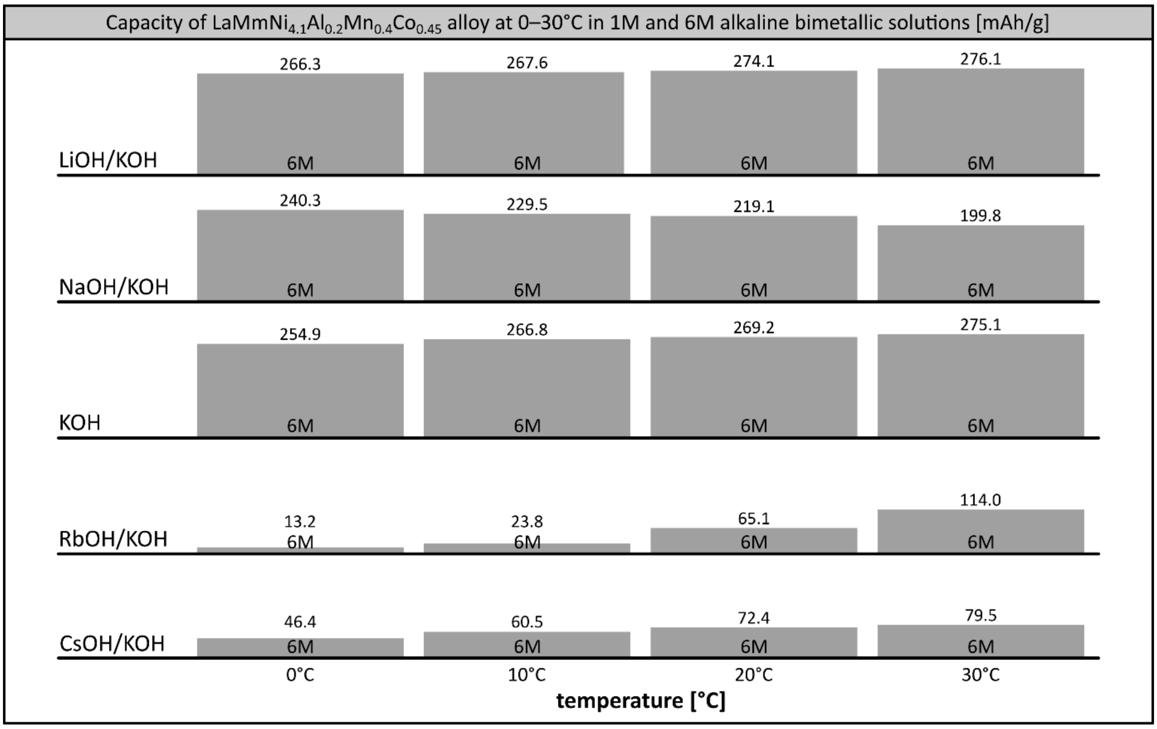

Figure S10. Electrochemical capacity of LaMm–$Ni_{4.1}Al_{0.3}Mn_{0.4}Co_{0.45}$ alloy as a function of composition of 6M MOH/KOH (M = Li–Cs) solutions at temperatures in the rang 0–30 °C in a series of decreasing temperature [M. Karwowska et al., Electrochim. Acta., 252 (2017) 381].

4. SEM images of corrosion structures at the surface of LaMm-$Ni_{4.1}Al_{0.3}Mn_{0.4}Co_{0.45}$ alloy [1/5]

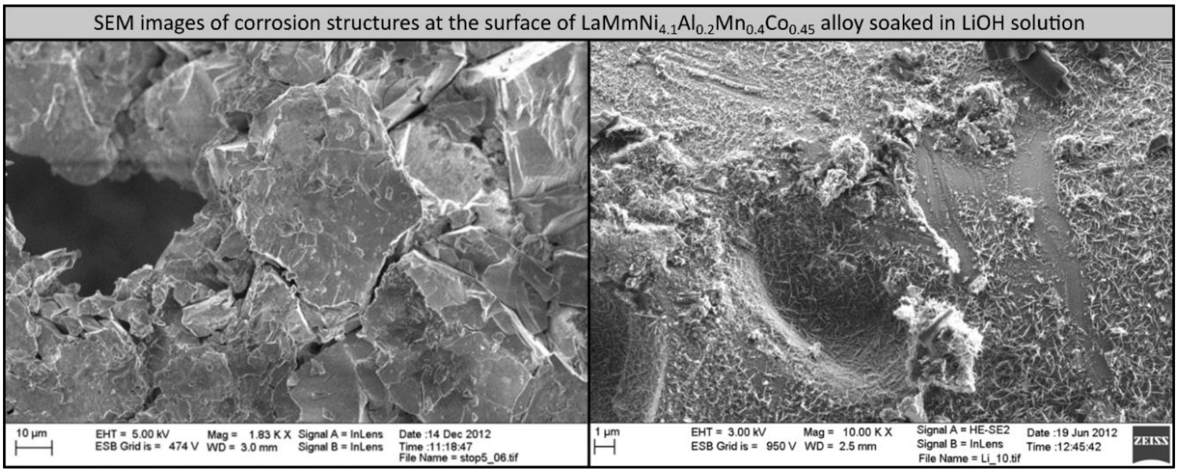

Figure S11. SEM images of $LaMmNi_{4.1}Al_{0.2}Mn_{0.4}Co_{0.45}$ alloy electrochemically treated in 1M LiOH at 30 °C. [M. Karwowska et al., Materials, 11 (2018) 2423].

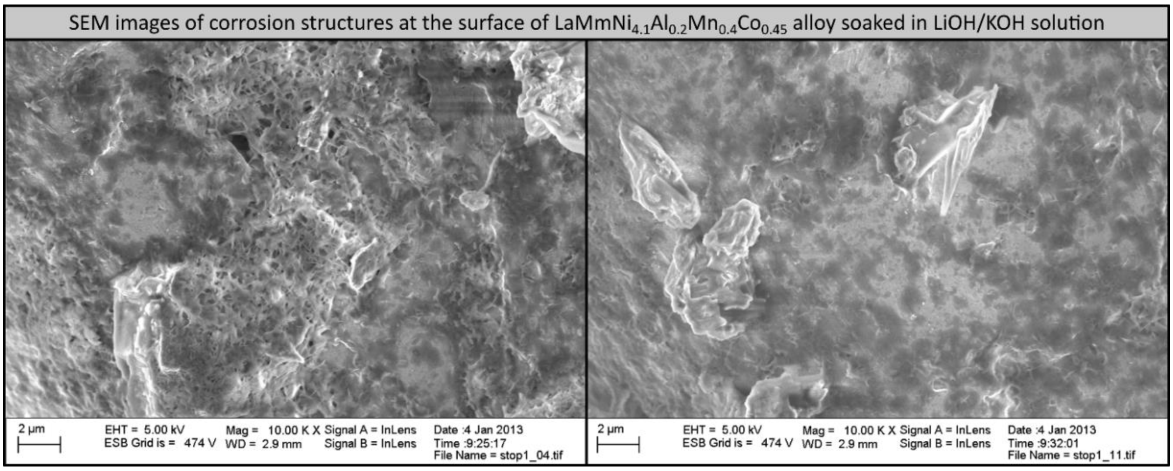

Figure S12. SEM images of $LaMmNi_{4.1}Al_{0.2}Mn_{0.4}Co_{0.45}$ alloy electrochemically treated in 6M LiOH/KOH at 30 °C. [M. Karwowska et al., Materials, 11 (2018) 2423].

4. SEM images of corrosion structures at the surface of LaMm-$Ni_{4.1}Al_{0.3}Mn_{0.4}Co_{0.45}$ alloy [2/5]

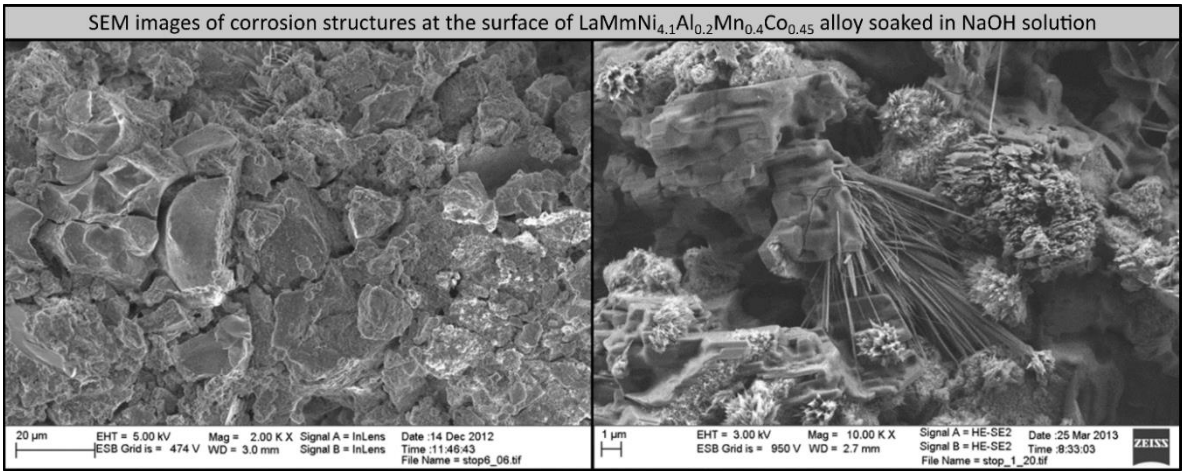

Figure S13. SEM images of $LaMmNi_{4.1}Al_{0.2}Mn_{0.4}Co_{0.45}$ alloy electrochemically treated in 6M NaOH at 30 °C. [M. Karwowska et al., Materials, 11 (2018) 2423].

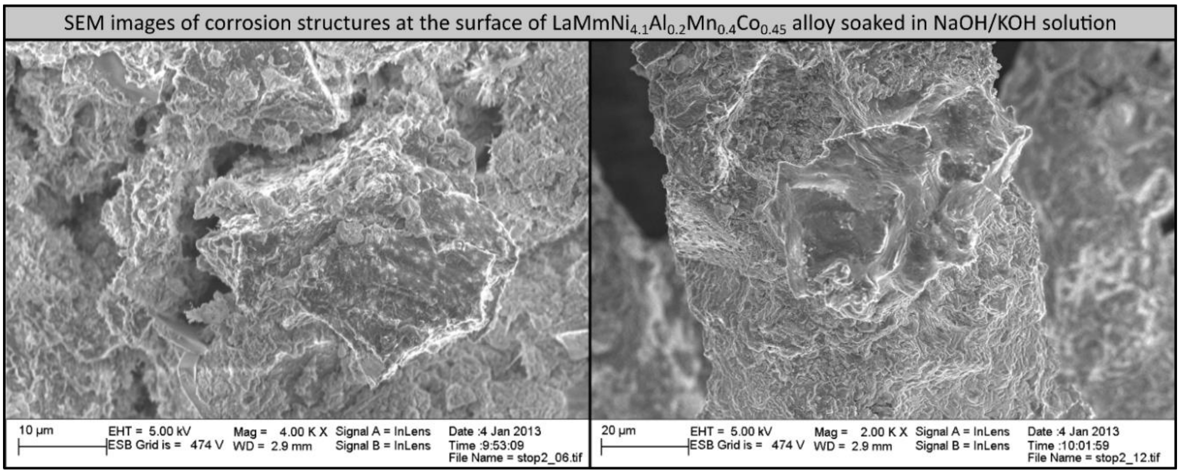

Figure S14. SEM images of $LaMmNi_{4.1}Al_{0.2}Mn_{0.4}Co_{0.45}$ alloy electrochemically treated in 6M NaOH/KOH at 30 °C. [M. Karwowska et al., Materials, 11 (2018) 2423].

4. SEM images of corrosion structures at the surface of LaMm-$Ni_{4.1}Al_{0.3}Mn_{0.4}Co_{0.45}$ alloy [3/5]

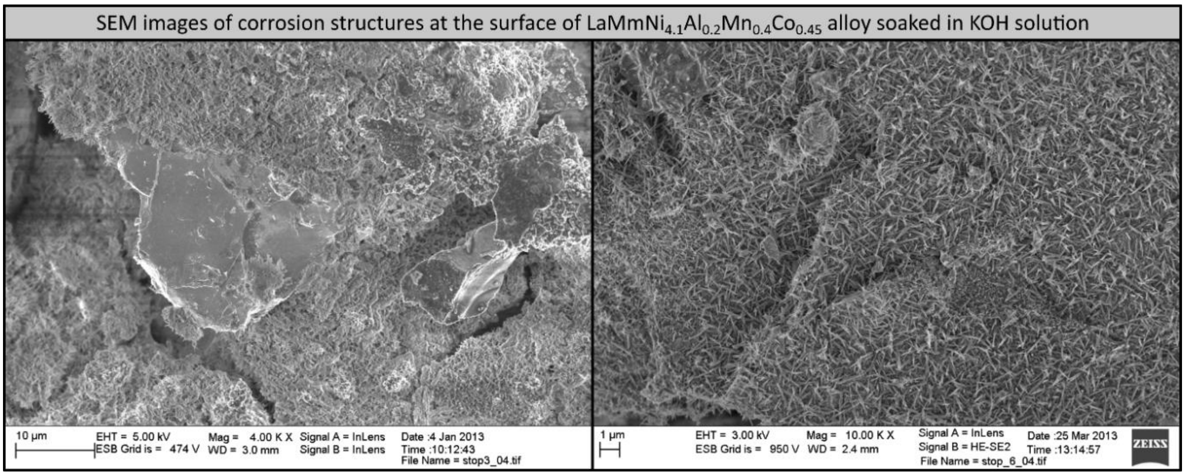

Figure S15. SEM images of $LaMmNi_{4.1}Al_{0.2}Mn_{0.4}Co_{0.45}$ alloy electrochemically treated in 6M KOH at 30 °C. [M. Karwowska et al., Materials, 11 (2018) 2423].

4. SEM images of corrosion structures at the surface of LaMm-$Ni_{4.1}Al_{0.3}Mn_{0.4}Co_{0.45}$ alloy [4/5]

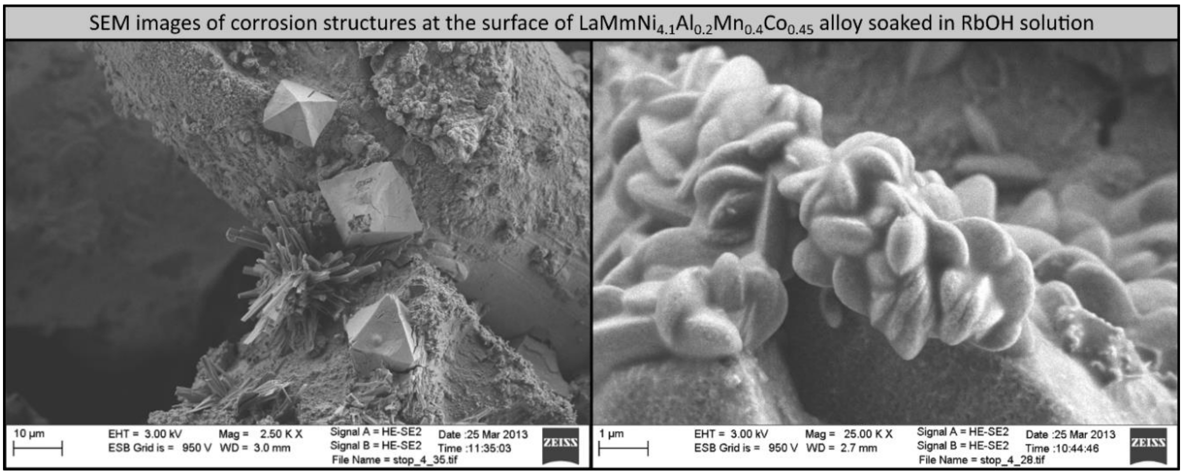

Figure S16. SEM images of $LaMmNi_{4.1}Al_{0.2}Mn_{0.4}Co_{0.45}$ alloy electrochemically treated in 6M RbOH at 30 °C. [M. Karwowska et al., Materials, 11 (**2018**) 2423].

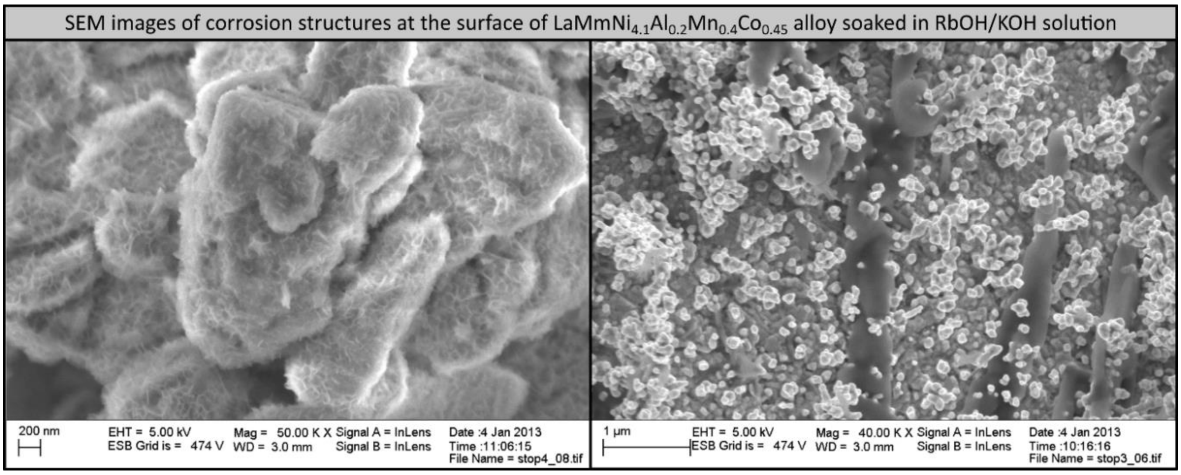

Figure S17. SEM images of $LaMmNi_{4.1}Al_{0.2}Mn_{0.4}Co_{0.45}$ alloy electrochemically treated in 6M RbOH/KOH at 30 °C. [M. Karwowska et al., Materials, 11 (2018) 2423].

4. SEM images of corrosion structures at the surface of LaMm-$Ni_{4.1}Al_{0.3}Mn_{0.4}Co_{0.45}$ alloy [5/5]

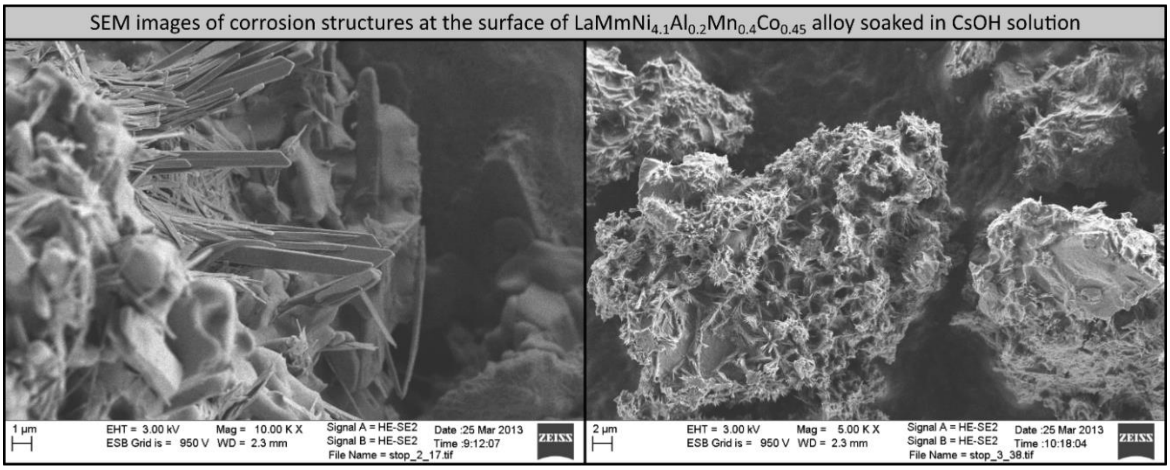

Figure S18. SEM images of $LaMmNi_{4.1}Al_{0.2}Mn_{0.4}Co_{0.45}$ alloy electrochemically treated in 6M CsOH at 30 °C. [M. Karwowska et al., Materials, 11 (2018) 2423].

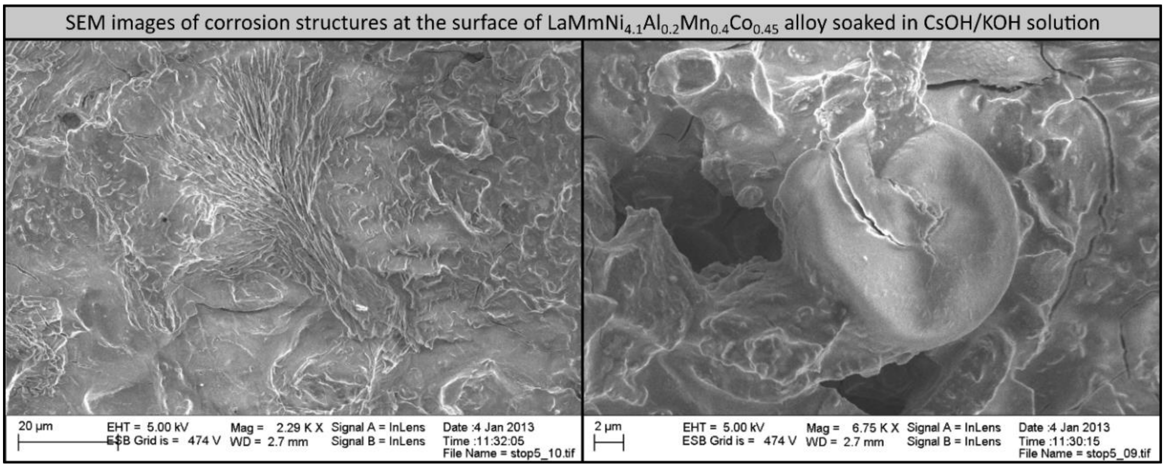

Figure S19. SEM images of $LaMmNi_{4.1}Al_{0.2}Mn_{0.4}Co_{0.45}$ alloy electrochemically treated in 6M CsOH/KOH at 30 °C. [M. Karwowska et al., Materials, 11 (2018) 2423].

© 2019 by the authors. Submitted for possible open access publication under the terms and conditions of the Creative Commons Attribution (CC BY) license (http://creativecommons.org/licenses/by/4.0/).
